# Chlamydia trachomatis prevalence in undocumented migrants undergoing voluntary termination of pregnancy: a prospective cohort study

**DOI:** 10.1186/1471-2458-8-391

**Published:** 2008-11-24

**Authors:** Hans Wolff, Ana Lourenço, Patrick Bodenmann, Manuella Epiney, Monique Uny, Nicole Andreoli, Olivier Irion, Jean-Michel Gaspoz, Jean-Bernard Dubuisson

**Affiliations:** 1 Department of Community Medicine and Primary Care, University Hospitals of Geneva, University of Geneva, Switzerland; 2 Department of Obstetrics and Gynaecology, University Hospitals of Geneva, University of Geneva, Switzerland; 3 Department of Ambulatory Care and Community Medicine, University of Lausanne, Switzerland

## Abstract

**Background:**

Chlamydia trachomatis infection (CTI) is the most frequent sexual transmitted disease (STI) in Switzerland but its prevalence in undocumented migrants is unknown. We aimed to compare CTI prevalence among undocumented migrants undergoing termination of pregnancy (ToP) to the prevalence among women with residency permit.

**Methods:**

This prospective cohort study included all pregnant, undocumented women presenting from March 2005 to October 2006 to the University hospital for ToP. The control group consisted of a systematic sample of pregnant women with legal residency permit coming to the same hospital during the same time period for ToP

**Results:**

One hundred seventy five undocumented women and 208 women with residency permit (controls) were included in the study. Mean ages were 28.0 y (SD 5.5) and 28.2 y (SD 7.5), respectively (p = 0.77). Undocumented women came primarily from Latin-America (78%). Frequently, they lacked contraception (23%, controls 15%, OR 1.8, 95% CI 1.04;2.9). Thirteen percent of undocumented migrants were found to have CTI (compared to 4.4% of controls; OR 3.2, 95% CI 1.4;7.3).

**Conclusion:**

This population of undocumented, pregnant migrants consisted primarily of young, Latino-American women. Compared to control women, undocumented migrants showed higher prevalence rates of genital CTI, which indicates that health professionals should consider systematic screening for STI in this population. There is a need to design programs providing better access to treatment and education and to increase migrants' awareness of the importance of contraception and transmission of STI.

## Background

Geneva (Switzerland), as one of the wealthiest areas of the world, is a common target of illegal migration. Most of these undocumented migrants, also called "illegals" or "clandestines", leave their home country because of difficult economic conditions. An estimated 8,000 to 12,000 undocumented migrants, who lack legal residential permit, live and work in the canton of Geneva [1], representing 1.4 to 3.5% of the 434,500 resident population. Because of their difficult living conditions, separation from their families, permanent threat of being caught by the police, and exclusion from the health care system, it is reasonable to believe that undocumented migrants are in poor health. Since undocumented migrants do not have any social protection, unintended pregnancy may place them at risk when associated with loss of work and income. Previous studies of undocumented migrants delivering in Geneva found a high rate of unintended pregnancies (75 to 83%) and inadequate contraceptive use [2,3].

Chlamydia trachomatis infection (CTI) is the principal cause of sexually transmitted infections (STI) in the developed world [4]. CTI is frequently asymptomatic but may have serious sequelae if untreated, such as chronic pelvic pain, ectopic pregnancy, and pelvic inflammatory disease with subsequent risk of infertility.

Surveillance data based on declaration of the Swiss national laboratory reports showed an increase in the number of CTI diagnosed by approximately 50% between 1999 and 2005 [5]. Nevertheless, the precise burden of disease remains unclear. A prevalence study among Swiss gynaecologists including 772 sexually active women found a CTI prevalence of 2.8% [6]. This study revealed an important degree of underreporting by the national laboratory reports, which are the only source of national data on CTI in Switzerland. In Europe, CTI prevalence is estimated at 3.4% in asymptomatic women [7] and 5 to 12% for women undergoing termination of pregnancy (ToP) [7-9]. Studies in Latin America show CTI prevalence rates of 1.9% to 4.5% in Chile, Peru, Brazil, and Mexico [4,10,11] and 12.2% in women attending family planning clinics [4,12].

The aim of the present study is to compare the prevalence of CTI in women with and without legal residency permit undergoing ToP in Geneva, Switzerland.

## Methods

### Voluntary ToP in Switzerland

In Switzerland, termination of pregnancy up to 12 weeks after onset of amenorrhea is available at women's request when performed by a trained gynaecologist in an authorised public or private clinic. ToP must be declared anonymously to health authorities and medical expenses are covered by general mandatory health insurance.

### Health care facility offering free access to health care

Since 1996, a health care facility offers medical care for free or at low cost to undocumented migrants in Geneva. It has no formal administrative requirements and has a high visibility among the local migrant population, reaching the majority of pregnant, undocumented, and uninsured women [13]. The health care facility refers all undocumented women to the Woman's University Hospital where they can receive free care.

### Study plan and population

#### Undocumented migrants

Between March 2005 and October 2006, this prospective cohort study included all undocumented pregnant women requesting ToP and presenting to the Woman's University Hospital, which is the only public woman's hospital in Geneva. In 2003, 74% of the 1,413 ToP's in Geneva were performed in this hospital. All women requesting ToP, including those presenting directly to the hospital, met with two coordinating nurses.

#### Control group

The control group consisted of a systematic sample of women with legal residency permit and mandatory health insurance undergoing ToP at the Woman's University Hospital. The undocumented migrants and the control group were assigned to the same nurses. Patient sampling occurred during predetermined days, from November 2005 to May 2006. In each pre-selected day, all women were asked to be included in the study.

#### Exclusion criteria

We excluded women who could not communicate in French, Spanish, or English. Women attending the free health care facility but not considered to be undocumented migrants (e.g. tourists) were excluded.

#### Questionnaire and blood tests

Two specially trained nurses speaking all three study languages administered a socio-demographic questionnaire in a face-to-face interview. The questionnaire included 21 questions about age, health insurance, nationality, education, civil status, duration of stay in Geneva, and contraceptive methods. CTI was assessed by polymerase chain reaction (PCR) performed on cervical swabs.

#### Ethical considerations

All undocumented migrants and controls gave written consent. The study was approved by the ethical research committee of the Geneva University Hospitals (no 05058-CD).

#### Main outcomes and potential confounders

The main study outcome was CTI. The main potential confounding factors were considered to be age, Latin American origin, civil status, and education.

### Statistical analysis

In order to investigate the relationship between legal status and the main outcome, we first used 2 × 2 tables and performed Chi-square and Fisher's exact tests to compare proportions for categorical variables and unpaired student's t-tests to compare means for continuous variables. We performed the same analyses after stratifying for potential confounding factors. Finally we used multiple logistic regression analysis adjusting systematically for age and those confounding factors that remained statistically significant (p < 0.05) in the models. All analyses were performed using SPSS for Windows (version 15.0).

### Missing data

Missing data are indicated in the tables. Only those study participants who had relevant complete data were included in the statistical analyses.

## Results

**Figure 1:**
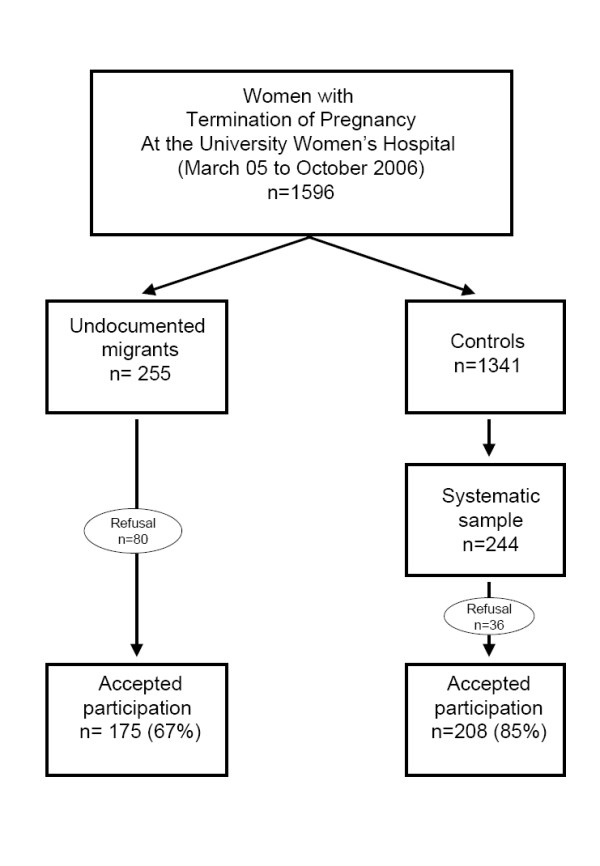
Flow-chart illustrating the selection of undocumented migrants vs. women with legal residency status (control group) who had voluntary termination of pregnancy (ToP) at the Geneva University Hospital, Switzerland between March 2005 and October 2006.

During 20 months (from March 2005 to October 2006), 1596 adult women underwent ToP at the Woman's University Hospital, of which 255 were undocumented migrants. One hundred seventy five (67%) accepted participation in the study, and 208 (85%) of 244 controls were selected for the study. As illustrated in Figure 1, 80 undocumented women (33%, mean age 32.3 years, range 17–47) and 36 controls (15%, mean age 29.4 years, range 18–46) refused participation, mainly because of lack of time.

### Socio-demographic characteristics

The socio-demographic characteristics are summarized in Table 1. Age was similar in both study groups (28 years, p = 0.77). Seventy-eight percent of the undocumented women came from Latin America and had lived in Geneva for approximately 4 years. The control group was primarily composed of Europeans (82%). Years of education were similar in both groups (12.3 vs 12.8 years, p = 0.19).

**Table 1:** Sociodemographic characteristics of undocumented migrants vs. women with legal residency status (control group) who had voluntary termination of pregnancy (ToP) at the Geneva University Hospitals, Switzerland between March 2005 and October 2006

Characteristic or measurement	Undocumented (n = 175)	Missing n (%)	Control group (n = 208)	Missing n (%)	p-value
Mean age in years (SD)	28.0 (5.5)	0	28.2 (7.5)	0	0.77
Continent of origin (%)	Latin America 78.2, Europe 0.6, Asia 8.0, Africa 13.2	1 (0.6)	Europe 82.0, Lat.-America 7.3, Africa 8.7, Asia 1.5, Australia 0.5	2 (1.0)	<0.001
Nationality (%)	Bolivia 43.3, Brazil 13.7, Equator 9.1, Cameroun 6.3, Mongolia 4.0, Philippines 2.3, Colombia 2.9	1 (0.6)	Switzerland 51.9, Portugal 11.1, France 5.8, Brazil 3.4, Spain 2.9	2 (1.0)	<0.001
Civil status:					<0.001
Single	69.5%	1 (0.6)	79.9%	6 (2.9)	
Married	17.2%	1 (0.6)	19.8%	6 (2.9)	
Divorced	12.6%	1 (0.6)	0.5%	6 (2.9)	
Widowed	0.6%	1 (0.6)	0%	6 (2.9)	
Education:					
Education years (SD)	12.3 (3.3)	12 (6.9)	12.8 (3.5)	2 (1.0)	0.19
Highest achieved education:					0.006
Primary school	5.5%	12 (6.9)	1.5%	2 (1.0)	
Secondary school	40.5%	12 (6.9)	56.3%	2 (1.0)	
High school degree	34.4%	12 (6.9)	24.3%	2 (1.0)	
University	19.6%	12 (6.9)	18.0%	2 (1.0)	
Years living in Geneva (SD)	3.8 (7.4)	4 (2.3)	14.8 (10.6)	3 (1.4)	<0.001

### Contraception and ToP

As shown in table 2, undocumented migrants had their first contact with a health professional one week later than controls (8.0 vs. 6.9 weeks of pregnancy, p < 0.001). Insecure contraceptive methods (condoms, withdrawal, calendar) were frequent in both study groups, used by 83.6% of the undocumented migrants and 75.7% of the controls. No difference between the study groups could be observed concerning condom use and withdrawal (coitus interruptus). However, undocumented migrants more frequently used the calendar method (29.9% vs 17%, OR 2.2, 95% CI 1.3;3.8). Furthermore, compared to controls, almost twice as many undocumented women did not use any contraceptive method (OR 1.8, CI:1.0;2.9), and only half of them used the contraceptive pill during the month of conception (OR 2.1, 95% CI 1.1;3.8). Stated reasons for absence of contraception in undocumented migrants were: infrequent intercourse, 22%; short term contraception interruption (running out of pills, pill side effects, or lack of money to buy pills), 20%; belief of being infertile, 16%; partner had refused to use condoms, 10%; lacking knowledge on contraceptive methods, 6% ; and not knowing where to get contraception, 6%. Other reasons noted to explain the absence of contraception were: "I didn't think about it", "latent wish of pregnancy but planned for later", and "presumed sterility of the partner".

**Table 2:** Access to care, contraception, chlamydia trachomatis infection, history and type of voluntary pregnancy interruption (ToP) of undocumented pregnant migrants vs. pregnant women with legal residency status (control group) who had ToP at the Geneva University Hospitals, Switzerland between March 2005 and October 2006

Characteristic or measurement	Undocumented migrants (n = 175)	Missing n(%)	Control group (n = 208)	Missing n(%)	Mean difference (CI95%)° or OR (CI95%)°°
Gravidity (SD)	2.8 (1.6)	1 (0.6)	2.4 (1.4)	6 (2.9)	0.4 (0.1;0.7)°
Parity (SD)	1.2 (1.2)	0	0.8 (0.9)	0	0.4 (0.2;0.6)°
Weeks of pregnancy at first contact with a health professional (SD)	8.0 (2.6)	0	6.9 (2.1)	0	1.2 (0.7;1.6)°
Delayed first contact with a health professional					
(>10 weeks of amenorrhea)	14.9%	0	5.8%	0	2.9 (1.4;5.8)°°
(>12 weeks of amenorrhea)	4.0%	0	1.4%	0	2.9 (0.7;11.7)°°
Chlamydia trachomatis infection: (CTI)	12.8%	3 (1.7)	4.4%	2 (1.0)	3.2 (1.4;7.3)°°
CTI by type of contraception:					
Condom use	7.0%	3 (1.7)	3.8%	2 (1.0)	1.9 (0.4;9.0)°°
Other means of contraception	15.7%	3 (1.7)	4.8%	2 (1.0)	3.7 (1.4;9.7)°°
Contraception the month of conception:					
No contraception	23.4%	0	14.9	0	1.8 (1.04;2.9)°°
Insecure contraception (of those with contraception)	83.6%	0	75.7%	0	1.6 (0.9;2.9)°°
Condom	42.5%	0	46.3%	0	1.4 (0.9;2.1)°°
Calendar (Ogino)	29.9%	0	17.0%	0	2.2 (1.3;3.8)°°
Withdrawal	11.2%	0	12.4%	0	1.1 (0.6;2.3)°°
Secure contraception (of those with contraception):					
Pill	12.7%	0	21.5%	0	2.1 (1.1;3.8)°°
Others	3.7%	0	2.8%	0	1.6 (0.4;5.8)°°

°Mean difference for continuous data (if value 0 included in CI95% then p > 0.05),

°°Age-adjusted OR for categorical data (if value 1 included in CI95% then p > 0.05)

### Chlamydia trachomatis infection (CTI)

CTI was three times more frequent in undocumented migrants (12.8%) than in controls (4.4%, crude OR 3.2 (95% CI 1.4;7.2)). The OR adjusted for age was 3.2 (95% CI 1.4;7.3).

Stratification by origin (Latin American vs. not of Latin American origin) showed a similar increase of CTI prevalence among undocumented migrants: 5.8% (95% CI -9.9;21.5) vs.6.7% (95% CI -2.7;16.2).

## Discussion

The prevalence of genital CTI in women requesting ToP was threefold higher in undocumented migrants than in women with legal residency permit (OR 3.2 (95% CI 1.4;7.3)). Insecure contraceptive methods (condoms, withdrawal, calendar) were frequent in both study groups. Compared to the control group, undocumented migrants used less hormonal contraception and were twice as likely to not use any contraception. The lesser use of contraception and the fact that over one third of women reported to have occasional partners may partially explain the high prevalence of CTI (12.8%) in this group.

There are no population studies on CTI prevalence in Switzerland. However, data from sentinel populations and laboratory findings have shown an overall prevalence of CTI of 2.8 to 4% [6,14], which corresponds to the findings in our control group (4.4%) and is three times lower than the prevalence found here in undocumented migrants (12.8%). In a recent study conducted in women screened for ToP in Liverpool, the prevalence of CTI was 7.3%, mostly affecting women from 20 to 24 years of age [15]. Studies in Latin America show CTI prevalence rates of 1.9% to 4.5% in Chile, Peru, Brazil, and Mexico [4,10,11] and a rate of 12.2% in women attending family planning clinics [4,12]. A study in Italy offered free screening for STI to female migrant sex workers and found a CTI prevalence of 14% [16]. The highest prevalence rates were observed in sex workers from Eastern Europe and, although not directly comparable, it suggests that undocumented migrant women in our study may actually be engaging in high risk sexual behaviours, whether on a voluntary basis or not. In any case, the encountered high prevalence rates of CTI and the availability of single-dose antibiotic treatment urges health professionals to consider systematic STI screening.

To the best of our knowledge, this is the first time in a Europe, that ToP characteristics were studied in undocumented migrants and compared to a local control group. Another strength of the study is the prospective and systematic inclusion of a relatively large number of this hard-to-reach population.

Limitations of the current study concern the representativeness of our sample from this hard-to-reach population and from the general population of pregnant women. Nevertheless, several aspects lead us to believe that this study reached a substantial proportion of pregnant, undocumented women requesting ToP in Geneva and is therefore representative of them: 1) The free medical care unit is well known by this hard-to-reach population; 2) The proportion of Latin Americans (78%) is similar to that found by other sources: by investigation of the origin of undocumented workers, the Geneva trade union recently found 76% were Latin Americans [17]; 3) In order to achieve optimal participation, undocumented women were enrolled in collaboration with the Woman's University Hospital, which is the only public Women's hospital in the Canton of Geneva. Finally, it is possible that the study sample still differs from the whole undocumented population of Geneva, which is, by definition, unknown. Although our control group was not a random sample from the general population, it was obtained by systematically sampling all the women with valid residence permits who were seen on selected days at the same hospital by the same nurse during the same time period that the sample of undocumented migrants was obtained.

## Conclusion

This population of undocumented, pregnant migrants consisted primarily of young, Latino-American women. Compared to control women, undocumented migrants showed higher prevalence rates of genital CTI, which indicates that health professionals should consider systematic STI screening. There is a need for programs providing better access to treatment and education and to increase the migrants' awareness of the importance of contraception and transmission of STIs.

## Abbreviations

CTI: chlamydia trachomatis infection; STI: sexually transmitted infection; CI: confidence interval; OR: odds ratio; SD: standard deviation; ToP: voluntary termination of pregnancy.

## Competing interests

The authors declare that they have no competing interests.

## Authors' contributions

HW conceived the study, participated in its design and coordination, performed the statistical analysis, interpretation of the data, and drafted the manuscript. APL and ME participated in the study design, coordination, interpretation of the data, and gave critical contribution to the manuscript. PB participated in the interpretation of the data and gave critical contribution to the manuscript. MU and NA contributed substantially to the acquisition of data. OI and JMG gave critical contribution to the manuscript and JBD participated in the study design and coordination and gave critical contribution to the manuscript. All authors read and approved the final manuscript.

## Pre-publication history

The pre-publication history for this paper can be accessed here:


